# First direct evidence of sedimentary carbonate recycling in subduction-related xenoliths

**DOI:** 10.1038/srep11547

**Published:** 2015-06-23

**Authors:** Yongsheng Liu, Detao He, Changgui Gao, Stephen Foley, Shan Gao, Zhaochu Hu, Keqing Zong, Haihong Chen

**Affiliations:** 1 State Key Laboratory of Geological Processes and Mineral Resources, School of Earth Sciences, China University of Geosciences, Wuhan, 430074, China; 2 ARC Centre of Excellence for Core to Crust Fluid Systems, Dept. of Earth and Planetary Sciences, Macquarie University, North Ryde, New South Wales 2109, Australia

## Abstract

Carbon in rocks and its rate of exchange with the exosphere is the least understood part of the carbon cycle. The amount of carbonate subducted as sediments and ocean crust is poorly known, but essential to mass balance the cycle. We describe carbonatite melt pockets in mantle peridotite xenoliths from Dalihu (northern China), which provide firsthand evidence for the recycling of carbonate sediments within the subduction system. These pockets retain the low trace element contents and δ^18^O_SMOW_ = 21.1 ± 0.3 of argillaceous carbonate sediments, representing wholesale melting of carbonates instead of filtered recycling of carbon by redox freezing and melting. They also contain microscopic diamonds, partly transformed to graphite, indicating that depths >120 km were reached, as well as a bizarre mixture of carbides and metal alloys indicative of extremely reducing conditions. Subducted carbonates form diapirs that move rapidly upwards through the mantle wedge, reacting with peridotite, assimilating silicate minerals and releasing CO_2_, thus promoting their rapid emplacement. The assimilation process produces very local disequilibrium and divergent redox conditions that result in carbides and metal alloys, which help to interpret other occurrences of rock exhumed from ultra-deep conditions.

Carbon is a key element necessary for life and is critical for maintaining a habitable environment, but our understanding of the carbon cycle is far from complete. The concept of the carbon cycle was developed largely in the atmospheric sciences and biogeochemistry^1^,^2^; the solid Earth was recognized as having enormous reservoirs of carbon, but with sluggish exchange rates of little interest on the time scales of the climate system. However, it is now recognized that carbon fluxes between the surface and the inner Earth may have been underestimated greatly, and that well over 90% of the Earth’s carbon is stored in the deep Earth^3^.

Fluxes of carbon between the solid Earth and the exosphere have played an important role in modulating Earth’s atmosphere and climate over geological time scales^4^. Vast amounts of CO_2_ are emitted into the atmosphere by volcanism, which is thought to influence climate and weather^5^. In the return direction, CO_2_ in the atmosphere is absorbed by water and precipitated as carbonate and carbonated silicate^6^, and a proportion of the CO_2_ is released back into the atmosphere during the weathering of carbonate-bearing rocks and metamorphism^7^.

Over long periods of time, the CO_2_ present in carbonated oceanic basalts and sediments has probably been mostly recycled back into the Earth’s interior at subduction zones. The subduction of carbonate-rich sediments and altered oceanic crust is the most important process in balancing the Earth’s carbon budget^3^, and could result in major heterogeneities in the chemical and *f*O_2_ structure of the mantle. Depending on the thermal gradients of subduction zones, carbonate may suffer different fates^8^. Sedimentary limestone could be thermally decomposed in hot subduction zones to form regional CO_2_ emissions^8^,^9^. However, along cool geotherms little loss of CO_2_ will occur from carbonate-bearing marine sediments, so that most of the subducted carbonate will bypass the volcanic arc source region and proceed to greater depths. Evidence for this is found in coesite- and diamond-bearing marbles from orogenic belts that have experienced ultra-high pressure (UHP) metamorphism^10^, as well as in high-pressure experiments on carbonated pelite^11^ and eclogite^12^, which show the persistence of carbonate above the initial melting temperature. Mineral inclusions in diamonds from Juina, Brazil indicate that sediments and carbonates could have been subducted to the lower part of the transition zone or even the lower mantle^13^.

A few deeply subducted carbonates have been exhumed to the surface without having reacted with mantle peridotite; for example, marbles in the UHP orogenic belts^10^. Most others return to the surface in the form of melts passing through the mantle wedge. Sediments may become detached from the subducting slab and form buoyant diapirs at temperatures of 500–850 °C and depths >40 km below the arc^14^. When these diapirs rise through the overlying hot mantle wedge, they may undergo partial melting to form carbonatite or CO_2_-rich silicate melts.

Mantle rocks become progressively more reduced with depth due to the effect of pressure on ferric/ferrous equilibria, and the deeper parts – perhaps even the lower half – of the upper mantle may be metal saturated^15^,^16^. Carbonatite melts derived from deeply subducted lithosphere would therefore be unstable when infiltrating the mantle at depths greater than 250 kilometres and be reduced to immobile diamond, a mechanism termed “redox freezing”^17^. When carbon-enriched regions formed by ‘redox freezing’ later move upwards in packets of upwelling mantle, diamond will inevitably react with the Fe^3^^+^ leading to ‘redox melting’^18^, forming carbonatitic melts.

A shortcut in the long-term carbon cycle may be achieved if the assimilation of carbonate sediments in magmas is widespread. Recent petrological and geochemical evidence in favour of this process^19^,^20^ has led to a revival of interest after the original proposals had appeared discredited.

In this article we describe carbonatitic xenoliths from basalt which demonstrate that sedimentary limestone can be subducted to at least >120 km, and then quickly returned to shallow depths in the lithosphere mantle where they were sampled as carbonatitic melt with compositions little different to the original limestone. The speed of this process leads to the preservation of mineral indicators that allow this path to be described.

## Results

### Geologic setting and sample collection

The Central Asian Orogenic Belt (CAOB) was formed by closure of the long-lived Paleo-Asian Ocean in the late Permian^21^. The Neogene Dalihu volcanic field is located in the Inner Mongolia–Daxinganling Orogenic Belt, which is the eastern extension of the CAOB. The Paleo-Asian ocean plate was subducted southward beneath the North China Craton, resulting in an accretionary zone between the craton and the Solonker suture, which was accompanied by late Carboniferous subduction-related magmatism^22^.

**Figure 1:**
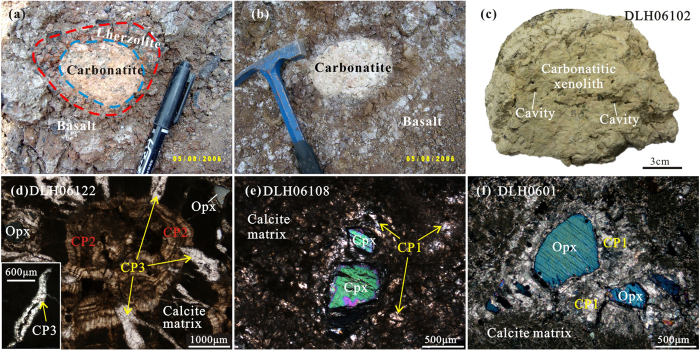
Field appearance of (**a**) carbonatitic xenolith with an envelope of metasomatized peridotite, and (**b**) carbonatitic xenolith exhibiting a sharp boundary with the host basalt. (**c**) Carbonatitic xenolith containing abundant irregular blebs/cavities on the scale of micrometers to millimetres. (**d**) Micrometer-sized cavities with an envelope of calcite phenocrysts. (**e**,**f**) Resorption of pyroxenes by carbonate demonstrated by recrystallized pseudomorphs of calcite after pyroxene. CP1 = Calcite phenocrysts distributed randomly or around the silicate minerals. CP2 = Radial calcite phenocrysts around the cavity. CP3 = Clean calcite phenocrysts in the veins cross cutting CP2.

The Dalihu volcanic field, where the lherzolite, pyroxenite and carbonatitic xenoliths were collected, occurs in this southern accretionary zone (SI Fig. 1). The host volcanic rocks have chemical compositions of Mg-rich alkaline basalt (42.5–44.9 wt% SiO_2_; 9.2–12.3 wt% MgO) with variable and high volatile (LOI = 2.0–6.7 wt.%) and Na_2_O + K_2_O contents (1.6–4.3 wt%). The lherzolite xenoliths, typically 2–8 cm in diameter, are composed of medium- to coarse-grained olivine (Ol) + clinopyroxene (Cpx) + orthopyroxene (Opx) + spinel ± calcite. Some xenoliths show features of reaction with a carbonatitic melt, including growth of additional pyroxene. The carbonatite xenoliths are round, 4–30 cm in diameter, and most exhibit a sharp boundary with the host basalt. Some are surrounded by an envelope of metasomatized peridotite too thick to have resulted from solid-state diffusion (Fig. 1a,b), demonstrating the coexistence of carbonatite with peridotite, indicating that the carbonatite existed in the form of quickly solidified melt pockets within the mantle.

### Petrology of the carbonatitic xenoliths

**Figure 2:**
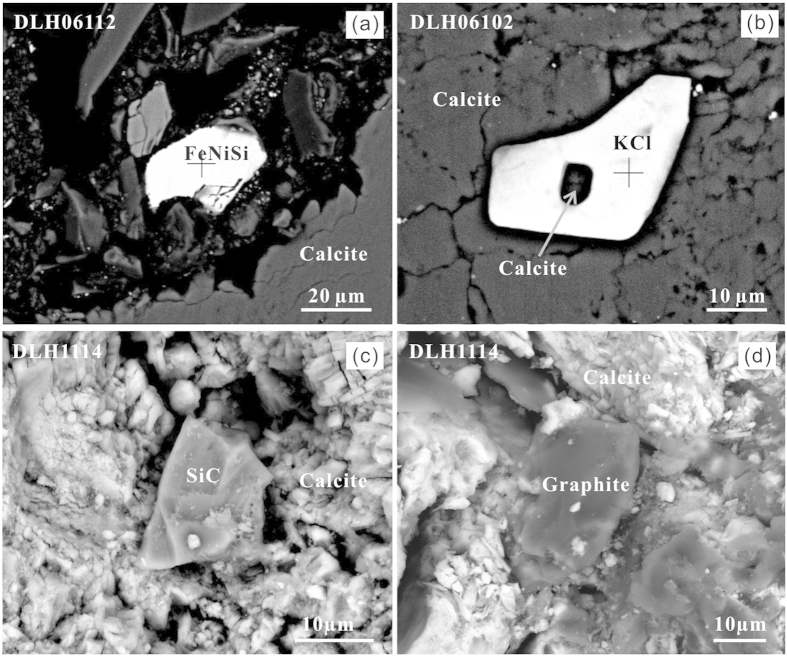
Alloy phase of FeNiSi (**a**) and potassium chloride (**b**) and moissanite [SiC] (**c**) and graphite (**d**) coexisting with carbonate.

**Figure 3:**
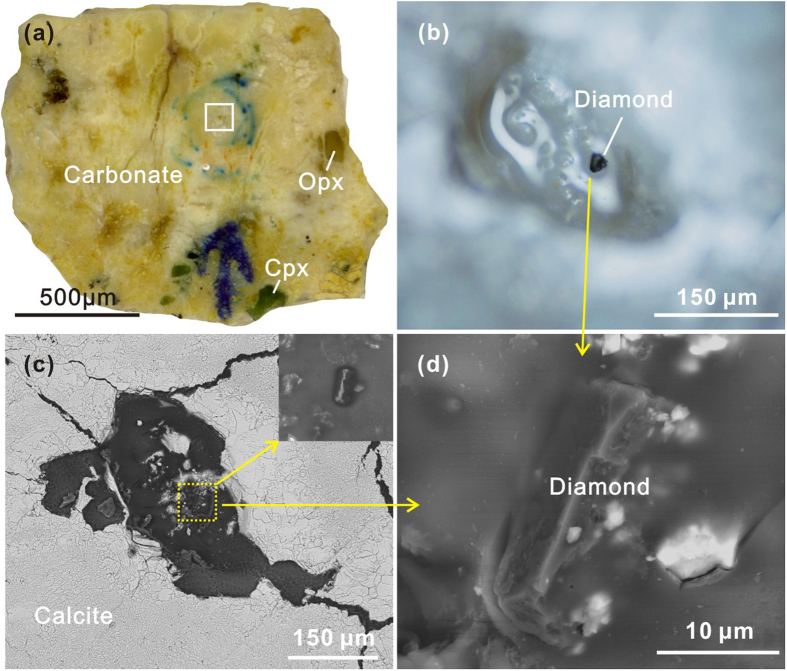
Occurrence of the diamond in carbonatitic xenolith. (**a**) Polished section of sample DLH1114. (**b**) Microphotograph, (**c**) SEM image, and (**d**) close-up SEM image of the diamond.

The carbonatitic xenoliths contain Opx, Cpx and calcite phenocrysts cemented by a fine-grained to cryptocrystalline carbonate matrix (Fig. 1d–f). Abundant irregular blebs, cavities or veins occur on the scale of micrometres to millimetres (Fig. 1c), which are generally surrounded or filled by calcite phenocrysts (Fig. 1d). The proportion of silicate minerals ranges from 1.5 to 15 vol.%, and the ratio of Opx/Cpx is 2 to 5. Crystals of feldspar (~20 μm), garnet (50–300 μm) and quartz (5–50 μm) were also identified. Rare tiny olivines (Fo = 91) were found in a few samples. Mg-Fe-silicate minerals were generally “resorbed” by carbonate as demonstrated by recrystallized pseudomorphs of calcite after pyroxene (Figs 1e,f). The carbonatitic xenoliths also contain small grains of diamond (approximately 20 μm), graphite (10–150 μm), moissanite (10–200 μm), titanium carbide (TiC; as inclusions in corundum), corundum (~50 μm), native metals (Si, Fe, Cu, Ni, Pt and Au), alloy phases (2–100 μm), pyrite and FeO (Figs 2 and 3). Graphite, moissanite, native metals and alloy phases were widely observed in the carbonatitic xenoliths.

Micro-diamond occurs in microcavities (150 × 100 μm) (Fig. 3) and was identified by its characteristic Raman peak at 1332 cm^−1^ (SI Fig. 2a). Although half of it is exposed, its surface is still about 10 μm below the surrounding carbonate matrix. Based on the Raman bands, the graphite can be classified into two types, highly ordered graphite with Raman bands at 1576–1581 cm^−1^ and disordered graphite with two bands at 1331 cm^−1^ and 1584 cm^−1^ (SI Fig. 2c,d). Moissanites in the polished sections (Fig. 2c) and as separated grains were identified by their characteristic Raman peaks at 763, 784 and 962 cm^−1^ and were generally found in the microcavities, although a few occur in the matrix carbonate. Moissanites may coexist with Au, Fe_3_Si, corundum and garnet. Separated moissanite grains are blue in color and display a generally metallic luster; transparent crystals with a brilliant sub-adamantine lustre are also present. A zircon inclusion was found in one moissanite grain (SI Fig. 2b) with unit-cell dimensions obtained with X-ray powder diffraction analysis on grains of a = 3.079 Å and c = 15.109 Å. Native silicon grains (5–80 μm) were found both in the microcavities and at boundaries between the calcite matrix and clinopyroxene grains (SI Table 1). The alloy phases vary significantly in stoichiometry but primarily consist of Fe_3_Si, Fe-Ni-Si and Fe-Cr (Fig. 2, SI Table 1). Potassium chloride crystals (KCl) were found in the calcite matrix and as inclusions in clinopyroxene, and one KCl crystal in the calcite matrix contains a calcite inclusion (Fig. 2b).

### Geochemical features of the carbonatitic xenoliths

**Figure 4:**
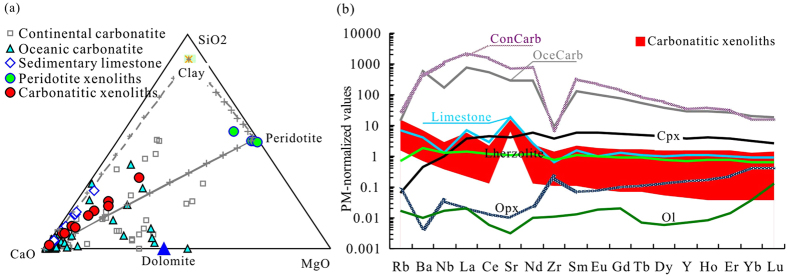
(**a**) Ternary SiO_2_-CaO-MgO diagram comparing carbonatitic and peridotite xenoliths to natural carbonatites and sedimentary carbonate rocks. (**b**) Primitive mantle (PM)-normalized trace element patterns of the carbonatitic xenoliths. Averages of sedimentary limestone^23^,^24^,^25^,^26^,^27^, continental (ConCarb) and oceanic carbonatite (OceCarb)^34^,^35^ are shown for comparison.

**Figure 5:**
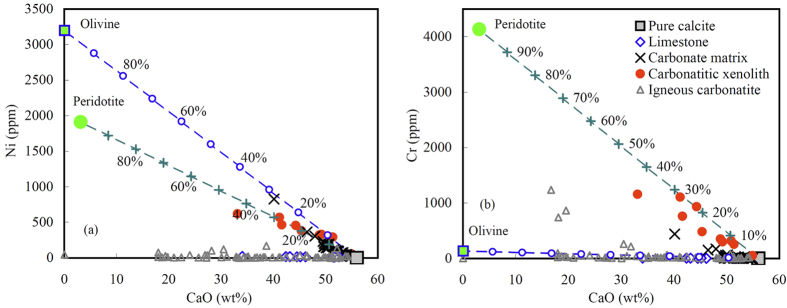
Plots of Ni and Cr versus CaO for the bulk carbonatitic xenoliths and carbonate matrix in the xenoliths. Limestone, igneous carbonatites and pure calcite are shown for comparison. Dashed lines are mixtures between peridotite or olivine and carbonate melt, numbers marked on the mixing lines are proportions of peridotite or olivine. Average of three peridotite xenoliths was used for the model calculation (SI Table 2).

The bulk compositions of the carbonatitic xenoliths show variable CaO/SiO_2_ ratios, forming a linear array to the SiO_2_ side of the CaO-peridotite join, similar to impure limestones with a significant argillaceous component. This is more reminiscent of sedimentary limestones than carbonatites (Fig. 4a), except that their compositions may be influenced by reaction with the surrounding peridotites. Trace element patterns are also similar to sedimentary carbonate rocks, with a remarkable positive Sr anomaly but generally low rare earth element (REE), large ion lithophile element (LILE) and high field strength element (HFSE) contents (Fig. 4b). The incompatible trace element contents of most samples are even lower than the average of sedimentary carbonate rocks^23^,^24^,^25^,^26^,^27^ (Fig. 4b). However, they have much higher Cr (51–1159 ppm) and Ni (56–620 ppm) contents than limestone (Cr = 5–37 ppm, Ni = 5–41 ppm^23^,^24^), and CaO contents show a strong negative correlation with Cr and Ni contents (correlation coefficients are −0.94 for Cr and −0.95 for Ni, respectively; Fig. 5). They have heavy oxygen isotopic compositions with δ^18^O_SMOW_ = 20.7–21.5.

Matrix carbonate in the xenoliths ranges from 72.1–98.0 wt.% CaCO_3_, 0.7–6.1 wt.% MgCO_3_ and 0.16–7.03 wt.% SiO_2_. They have relatively high Ni contents (20–827 ppm) and show a negative correlation between CaO and Ni (correlation coefficient = −0.95) (Fig. 5a). Almost all matrix carbonates have low Cr contents similar to limestone (Fig. 5b). Calcite phenocrysts range from 95.2 to 99.0 wt.% CaCO_3_, 0.5–4.3 wt.% MgCO_3_ and 0.15–0.56 wt.% SiO_2_, and have low Ni (<26.8 ppm) and Cr (<13.9 ppm) contents. Cpx and Opx in the carbonatitic xenoliths have similar chemical compositions to those in the lherzolite xenoliths (SI Table 2). The temperatures estimated from Cpx + Opx pairs^28^ in the carbonatitic xenoliths are 702–790 °C.

## Discussion

### Implications of the mineral assemblages in the carbonatitic xenoliths

The co-existence of diamond, ordered and disordered graphites provides potential records of pressure, temperature and redox conditions for the mantle source, whereas moissanite and native metals may track the emplacement conditions experienced by the xenoliths. Natural diamond generally forms at pressures >5-6 GPa and temperatures in the range of 900–1400 °C, but may form at depths as shallow as 120 km along subduction geotherms^29^. Experiments have shown that diamond formation via carbonate-silicate interaction can occur at pressures of 6-7 GPa and temperatures of 1350–1800 °C^30^. Thus, the preservation of tiny diamond crystals in the carbonatitic xenoliths indicates that they spent time at depths in excess of at least 120 km, and possibly 150 km. The D band of 1334 cm^−1^ (the typical Raman shift of diamond) in the disordered graphite most likely arises from the distortion of the diamond structure during its transformation to graphite^31^. This indicates that the ordered and disordered graphites distributed widely through the carbonatitic xenoliths are transformation products from diamond during the upward migration of the carbonatitic melt. Although some fine-grained garnets were found in the carbonatitic xenoliths, they are not equilibrated with the pyroxene and thus cannot be used to estimate pressure. The low temperature estimated from Cpx-Opx pairs in the carbonatitic xenoliths (702–790 °C) indicate interaction with carbonate melt, which is stable to about 150 °C lower than that of garnet-free peridotite (T = 820–1029 °C). Considering the coexistence with spinel lherzolite xenoliths (T = 784–1046 °C) and referring to the geotherm of the northern margin of NCC^32^, it appears that the carbonatitic melt pockets could have solidified and been stored in the shallow lithospheric mantle (40–60 km) for a short while before they were sampled and brought to the surface by the Neogene volcanics.

The carbonatitic xenoliths have much lower contents of most trace elements than do igneous carbonatites, which are characterized by enrichments in incompatible trace elements (e.g., Ba, Sr, Nb, Th, U and LREE) and steep light REE-enriched patterns^33^. Their trace element patterns also differ greatly from those of mantle-derived continental^34^ and oceanic carbonatites^35^, but are similar to sedimentary carbonate rocks (Fig. 4b). The heavy oxygen isotopic compositions (δ^18^O_SMOW_ = 20.7–21.5) fall in the range of Cretaceous to Palaeoproterozoic limestones (δ^18^O_SMOW_ = 17.5–30.9^36^), and are far removed from the δ^18^O_SMOW_ = 5–12 typical of igneous carbonatites^37^. In the SiO_2_-MgO-CaO plot, the carbonatitic xenoliths vary along a mixing trend between argillaceous limestone and peridotite (Fig. 4a). The melts that led to the crystallization of these xenoliths were primarily derived from argillaceous limestone subducted to at least 120 km rather than from partial melting of carbonated eclogites or peridotites.

Although CaO contents in the bulk xenoliths correlate well with Ni, Cr and CaO contents in the matrix, the proportion of carbonate correlates well with Ni, but not with Cr (Fig. 5). Both Ni and Cr are enriched in mantle peridotite but depleted in sedimentary carbonate rocks. The differing behaviour of Ni and Cr between the carbonatitic xenoliths and matrix carbonate confirms that (1) the carbonatitic xenoliths were formed by carbonate melt-peridotite interaction, rather than just olivine consumption, and (2) olivines were removed during the carbonate melt-peridotite interaction (probably partially replaced by pyroxene), as suggested by the absence of olivine in most carbonatitic xenoliths. Using average peridotite xenoliths and limestone as end members, mass balance calculations indicate that proportions of peridotite involved in the melt-peridotite interaction range from 1% to 30% (Fig. 5). Peridotite-melt interaction in the mantle wedge may have diluted REE, LILE, and HFSE, resulting in lower contents in the carbonatitic xenoliths than in sedimentary carbonate rocks (Fig. 4b). The compatibilities of REE, LILE and HFSE in clinopyroxene are much higher than those in olivine and opx, and the concentrations of these elements in olivine and opx are much lower than those in sedimentary carbonate rocks (Fig. 4b). Therefore, replacement of Ca-carbonate + olivine by Ca-Mg carbonate ± cpx ± opx is consistent with gradual depletion of these elements in carbonatitic melt. Unlike major and trace elements, δ^18^O_SMOW_ of the xenoliths cluster in a very small range (21.1 ± 0.3 [1σ, n = 9]) and are slightly lower than the grand average of sedimentary limestone (24.7 ± 3.0 (1σ, n = 102)). This implies (1) that oxygen isotopic diffusion within the carbonatitic melt is much quicker than that between the melt and peridotite, and (2) that the melt quickly solidified in the mantle so that only limited oxygen isotopic modification could occur by diffusion between peridotite and solidified carbonatitic melt pockets.

### The role of subducted carbonate in mantle processes above the subduction zone

**Figure 6:**
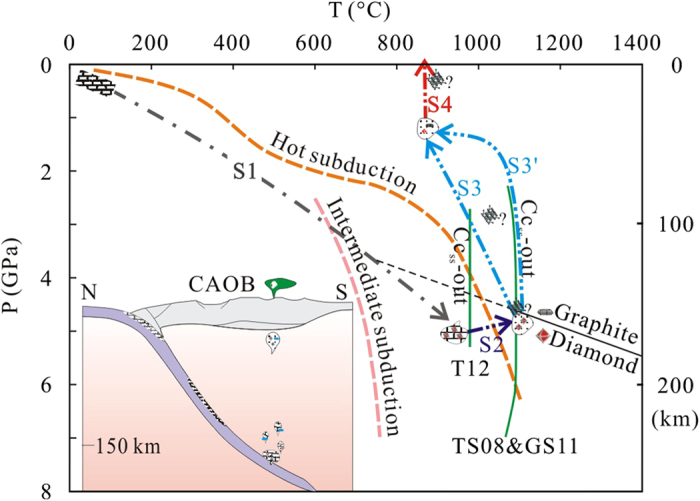
Deep mantle recycling of sedimentary limestone: S1 = subduction of crust with sedimentary limestone. S2 = diapirism of argillaceous limestone in the deep subduction zone (120–150 km) into the hot mantle wedge. S3 (or S3′) = rapid ascent of the carbonatitic melt. S4 = carbonated peridotite and carbonatitic xenoliths carried to the surface by the Neogene picrobasalt. The curves of hot subduction and intermediate subduction represent depth-temperature trajectories for the hottest and average slab-top conditions. Boundaries (Cc_ss_-out) for carbonated pelites from T12, 3–7 GPa^59^ and TS08, 2.5–5.0 GPa^11^ and GS11, ≥5.5 GPa^60^. The graphite–diamond reaction equilibrium is taken from Kennedy and Kennedy^61^.

Recycling of limestone into the deep mantle has been previously suggested based on the discovery of coesite- and diamond-bearing marbles from orogenic belts^10^,^38^,^39^. Furthermore, mineral inclusions in diamonds indicate that sediments and carbonates could have been subducted as deep as the upper part of the lower mantle^13^. Mantle rocks become progressively more reduced with depth and the lower reaches of the upper mantle may be metal saturated^15^,^16^. Diamond should be the dominant host for carbon at depths below 150 km, although carbides are also possible hosts in the deep mantle^40^. Thus, the carbonate in subducted limestone could potentially be partially reduced to form a carbon-saturated mixture with highly reduced phases when it moves into the deep mantle wedge (Fig. 6). Carbides crystallized under reducing conditions are known from polycrystalline diamonds, which are interpreted to have formed by reduction and redox freezing of carbonate melts^41^. KCl, which is found in the calcite matrix and as inclusions in clinopyroxene, can destabilize carbonate, allowing greater solubility and diffusion of carbon^42^, and is of great importance in forming diamonds^43^. The preservation of KCl in the carbonatitic xenoliths (Fig. 2b) indicates that the carbonatitic melt was saturated in halogens, which could have acted as a solvent catalyst for diamond growth.

Computed phase equilibria indicate that CO_2_-loss is negligible for carbonate-bearing marine sediments from depths of 80 to 180 km along low-temperature subduction geotherms^8^. Thomsen and Schmidt^11^ experimentally confirmed that >70–80% of the subducted carbonate will bypass the source region for volcanic arc melts and become transported to greater depths. This raises the question as to if and how the deeply subducted carbonate could be returned to the surface. Although deeply subducted carbonate could be tectonically exhumed to the surface as known from UHP orogenic belts, this may not account for melting to form carbonatites.

Rohrbach and Schmidt^17^ advocated a role for ‘redox freezing’ and ‘redox melting’ in the deep carbon cycle. If carbonatite melts were produced in deeply subducted lithosphere, they would be unstable when infiltrating the mantle wedge at depths greater than 250 kilometres and be reduced to diamond and thus immobilized. This redox freezing mechanism may form diamonds quickly, resulting in polycrystalline aggregates of diamond in which diamond is the major rock-forming mineral^41^,^44^. When such mantle packets, now carbon-enriched, upwell to higher levels, diamond will inevitably react with more oxidized mantle at shallower levels, oxidizing the diamond to carbonate and initiating redox melting to form deep-seated carbonatite melts. This coupled ‘redox freezing and melting’ process would transform the geochemical signature of the primarily REE-poor sedimentary carbonate into that of REE-rich carbonatite by low degree melting of carbonated peridotite^45^, because re-melting of the diamond aggregates together with surrounding peridotite on a larger scale equates to bulk melting of carbon-rich peridotite. This is a mechanism for transportation of carbon, but not for most of the other elements in the original carbonate rock. It may, however, be a precursor process for the formation of magmas seen at the surface as kimberlites and ultramafic lamprophyres^46^.

### Origin and emplacement of the carbonatitic xenoliths

These ‘redox freezing’ and ‘redox melting’ processes do not apply to the Dalihu carbonatite xenoliths, because the trace element and oxygen isotope characteristics of the limestone would not be transmitted to the carbonatite pods. Instead, they must have been produced by high-degree melting of argillaceous limestone. Although no data is available for the solidus of impure limestone at high pressures, the isobaric diagram for the system Na_2_CO_3_-CaCO_3_ at 1 kbar^47^ implies that the melting point of impure limestone is higher than the temperature at the surface of subducted slabs. Thus, limestones will not form high-degree melts at the top of the slab, but they may move in the form of buoyant diapirs due to their lower density than the surrounding peridotite. In other words, the mantle wedge likely undergoes carbonate fluxing by the diapiric rise of a marble mélange zone^14^ at temperatures below the melting point.

If the carbonate pockets migrate upward in the solid state, any diamonds they contain would inevitably be transformed to graphite. The survival of tiny diamonds and their disordered graphite breakdown products thus argues for *rapid* transportation into the shallow lithospheric mantle and cooling just shortly after segregation from the source. This would leave insufficient time for the complete elimination of diamond (Fig. 6). Melting of the carbonate-rich diapir probably occurred at high lithospheric levels during this process, leaving restricted time for assimilation and re-equilibration.

The abundant cavities and irregular blebs (Fig. 1c,d) may have formed by a volatile dissolution process similar to that suggested for the emplacement of kimberlites^48^. When carbonate diapirs rise through the overlying mantle wedge from the colder subduction zone, the combined effects of heating, decompression and SiO_2_ addition due to reaction of the carbonate with surrounding peridotite initiates partial melting, causing a catastrophic drop in the solubility of CO_2_, resulting in fluid expulsion^48^. This may facilitate rapid propagation of crack tips, promoting melt transportation in the lithospheric mantle, enhancing rapid and accelerating ascent of the carbonatitic melt into the shallow mantle, where it quickly cools to <800 °C as recorded by the Cpx + Opx pairs in the carbonatitic xenoliths. Melting of the diapir probably first occurred at high lithospheric levels, leaving restricted time for assimilation and re-equilibration.

### The origin of ultra-reduced phases

The origin of phases such as carbides and metal alloys in diamonds and kimberlites appear to indicate formation in extremely reducing conditions well below those typical of the upper mantle^49^,^50^. This has been controversial for many years. More recently, similar occurrences have been found in deeply subducted and exhumed ophiolites and serpentinites^51^,^52^. Since the maximum stability of moissanite in the upper mantle is several orders of magnitude *f*O_2_ below that for saturation in Ni-Fe metal^49^, and reduction of SiO_2_ to Si-metal requires even lower oxygen fugacities ( *f*O_2_ < IW-9 if αSiO_2_ = 1), appropriate conditions seem unattainable in the normal upper mantle^40^. The occurrence of carbides and alloys in the carbonatitic xenoliths requires either very local extremely reducing conditions, possibly related to vapor phase reactions^53^, or that the sedimentary carbonate rock was subducted into the lower half of the upper mantle where such reducing conditions may be prevalent before returning to the surface.

Under conditions of SiC stability, silicates must be essentially Fe-free due to the reduction of all Fe^2+^ to Fe, which is not observed. Carbonate melts, if present, should be rapidly reduced to diamond at depths greater than 250 km^17^, exhausting the melts. This appears to rule out large-scale equilibrium processes, favouring extremely local (millimetre-scale) redox variations in disequilibrium conditions at a late stage.

Shiryaev and Gaillard^53^ proposed that the extremely reducing conditions required for formation of moissanite, Si° and iron carbides may be achieved by SiC deposition from the gas phase at pressures <100 bars. However, this exact mechanism is difficult to envisage for the Dalihu rocks as it translates to a depth of less than 300 metres at 1300 °C. The host magma at the Dalihu locality is a picritic alkali basalt with low Mg/(Mg + Fe) of 0.58, indicating that some fractionation had occurred at upper mantle pressures. Experiments on a similar alkali-basalt from Skye indicate temperatures closer to 1200 to 1300 °C^54^, which would restrict the necessary strong reduction reactions to even closer to the surface or eliminate them completely.

However, KCl may have promoted and catalyzed the formation of carbides and metals on very local scales. Also, the reaction 3C + SiO_2_ → SiC + 2CO suggested by Shiryaev and Gaillard^53^ may be replaced by 2C + SiO_2_ → SiC + CO_2_ because CO concentrations are negligible at pressures ≥1 kbar^55^. SiO_2_ saturation is indicated by the presence of quartz and feldspar in the carbonatitic xenoliths.

There appear to be two possible scenarios for the formation of SiC and native Si: (1) they formed during rapid ascent of the carbonate-rich diapir from the top of the slab to the shallow mantle, which melted at a late stage *before* sampling by the basaltic melt; or (2) they formed during later transportation of carbonatitic xenoliths *within* the basaltic melt. We prefer the former because the massive degassing prompted by silicate assimilation^48^ could provide SiO_2_ and promote the above reaction, explaining the association of metal and carbide occurrences with cavities. Dusty inclusions of moissanite + quartz in diamonds from Fuxian kimberlite^56^ provide evidence for the same reaction in the deep mantle. Also, the breakdown of diamond to form two types of graphite would not have had time to occur in a process as close to the surface as 300 m.

Our work provides the first direct evidence for the recycling of sedimentary limestone to at least >120 km and for its rapid return through the mantle wedge in the form of a reactive carbonate-rich diapir and then carbonatite melt into the shallow lithosphere (Fig. 6). Such processes may have played an important role in changing the chemical composition of the mantle and in global carbon recycling since plate tectonics became operative.

## Methods

### Sample preparation

The carbonatitic xenoliths were pre-treated by immersion in a mixture of epoxy resin and curing agent in an evacuated environment to cement the minerals. To avoid possible contamination, the samples were then separately prepared by three methods for identifying different minerals: (1) *Polished sections for identifying diamond.* The surface of the carbonatitic xenolith was abraded to about 500 μm thickness by SiC abrasive papers of different sizes (600, 1000, 2000 mesh) to remove any possible exotic phases. Then, Al_2_O_3_ polishing with different grain sizes (5, 1, 0.3 μm) was used to polish sections progressively. (2) *Polished sections for identifying moissanite and graphite.* Al_2_O_3_ abrasive papers only were used to prepare the polished sections. The surface of the carbonatitic xenolith was firstly abraded about 1–2 mm thick by coarse Al_2_O_3_ abrasive papers (100 μm) to remove any possible exotic phases. Then, fine Al_2_O_3_ abrasive papers of different sizes were used to polish the sections progressively. (3) *Thin sections/polished sections for identifying phases other than diamond.* Diamond cutting blades, diamond abrasive papers and diamond polishing liquid only were used to make the sections.

### Raman spectroscopy, scanning electron microscope and electronic microprobe (CUG-Wuhan)

Diamond, SiC and graphite were analyzed using a Thermo Scientific DXR dispersive Raman micro-spectrometer. It was equipped with a 532 nm Nd-YVO4 laser, an automated confocal microscope (Olympus BX51) with a software-controlled x–y–z stage, and a Peltier-cooled charge-coupled device (CCD) detector. Frequencies of Raman bands were monitored by the 1001-cm^−1^ band of standard Polystyrene before and after each measurement, and the band-frequency accuracy was about 0.5 cm^−1^ (1σ level). All measurements were conducted at atmospheric pressure, room temperature (21 ± 1 °C) and humidity <50%.

Alloys and native metals were identified with a Quanta 200 environmental scanning electron microscope and GENSIS energy dispersive spectrometer using an accelerating voltage 20 kV, filament current 4.4nA, and working distance 11.5 mm. Major element compositions were then determined by a JEOL JXA-733 electron microprobe (EMP). Analyses were performed on polished thin sections using 15.0 kV accelerating voltage, 20 nA beam current and 1 μm beam diameter. Silicates and pure oxides were used as standards for calibration.

### XRF, ICP-MS and LA-ICP-MS analyses of bulk rocks and minerals

Whole rock samples were first crushed to finer than 5 mm in a corundum jaw crusher for bulk analysis. About 60 g was powdered to less than 200 mesh using a vibratory tungsten carbide disc mill. Major elements were analyzed by X-ray fluorescence (Rikagu RIX 2100) at Northwest University, China. Analyses of rock standards BCR-2 and GSR-3 indicate that both analytical precision and accuracy for major elements are generally better than 5%.

ICP-MS and LA-ICP-MS analyses were conducted at CUG-Wuhan. Bulk rock trace elements were analyzed by an Agilent 7500a ICP-MS About 50 mg samples were digested by HF + HNO3 in Teflon bombs for ICP-MS analysis. For detailed sample-digesting procedure for ICP-MS analyses see Liu *et al.*^57^. Detection limits for trace element analyses are listed in SI Table 2. Analyses of duplicate samples and rock standards BCR-2, BHVO-2, AGV-2 and RGM-2 indicate that precision of trace element analyses is generally better than 5% and accuracy is better than 10%.

Major and trace element analyses of minerals were conducted by LA-ICP-MS. Each analysis incorporated a background acquisition of approximately 20–30 s (gas blank) followed by 50 s of data acquisition from the sample. Element contents were calibrated against multiple-reference materials (BCR-2G, BIR-1G and BHVO-2G) without applying internal standardization. Analyses of MPI-DING reference glasses generally agree with recommended values within 5% for major elements, and 5–10% for trace elements. For details of operating conditions and data reduction see Liu *et al.*^58^.

## Additional Information

**How to cite this article**: Liu, Y. *et al.* First direct evidence of sedimentary carbonate recycling in subduction-related xenoliths. *Sci. Rep.* **5**, 11547; doi: 10.1038/srep11547 (2015).

## Supplementary Material

Supplementary Information
