# Supplementary material for: First direct evidence of sedimentary carbonate recycling in subduction-related xenoliths
Source: Sci Rep. 2015 Jun 23;5:11547. doi: 10.1038/srep11547 (PMC4477412; doi:10.1038/srep11547)

# First direct evidence of sedimentary carbonate recycling in subduction-related xenoliths

*Yongsheng Liu ^1^, Detao He ^1^, Changgui Gao ^1^, *Stephen Foley ^2^, Shan Gao ^1^, Zhaochu Hu ^1^, Keqing Zong ^1^, Haihong Chen ^1^

^1^ State Key Laboratory of Geological Processes and Mineral Resources, School of Earth Sciences, China University of Geosciences, Wuhan, 430074, China

^2^ ARC Centre of Excellence for Core to Crust Fluid Systems, Dept. of Earth and Planetary Sciences, Macquarie University, North Ryde, New South Wales 2109, Australia.

## Supplementary Information Table 1

Compositions of native metals, ferro-alloy, Fe-silicides and carbides (wt %)

Mineral Si Fe Ni Fe-Cr Fe3Si FeNiSi SiC TiC

____________________________________________________________________

Si 98.4 2.3 0.1 0.79 15.5 19.7 72.2 0.09

Ti 79.7

Al 0.02 0.20 0.4

Fe 0.12 96.8 0.2 70.4 83.6 61.1 0.03 0.04

Mn 0.17 0.52

Mg 0.05 0.01 0.21

Ca 0.46 1.6 0.11 1.2 0.19 0.14

Ni 0.01 98.7 8.1 0.54 20.2

Cu 0.01 2.7

Cr 0.02 0.01 16.7 0.16

_____________________________________________________________________

**Supplementary Information: Table 2**

Compositions of silicate minerals in the carbonatitic and lherzolite xenoliths

|  | Silicate minerals in carbonatitic xenoliths | | |  | Silicate minerals in lherzolite xenoliths | | |
| --- | --- | --- | --- | --- | --- | --- | --- |
|  | Ol | Opx | Cpx |  | Ol | Opx | Cpx |
| SiO_2_ | 40.79 | 56.04 | 53.87 |  | 41.44 | 54.52 | 52.56 |
| TiO­_2_ | 0.00 | 0.07 | 0.17 |  | 0.01 | 0.14 | 0.79 |
| Al_2_O_3_ | 0.00 | 2.41 | 3.26 |  | 0.00 | 3.67 | 5.82 |
| Cr_2_O_3_ | 0.00 | 0.52 | 1.28 |  | 0.00 | 0.34 | 0.81 |
| FeO | 8.74 | 5.53 | 2.35 |  | 9.29 | 6.40 | 2.46 |
| MnO | 0.01 | 0.14 | 0.10 |  | 0.12 | 0.15 | 0.05 |
| NiO |  | 0.08 | 0.06 |  | 0.36 | 0.11 | 0.04 |
| MgO | 49.58 | 34.40 | 16.58 |  | 49.36 | 34.66 | 14.74 |
| CaO | 0.60 | 0.76 | 21.51 |  | 0.01 | 0.36 | 21.51 |
| Na_2_O | 0.00 | 0.04 | 1.06 |  | 0.00 | 0.02 | 1.36 |
| K_2_O | 0.00 | 0.01 | 0.00 |  | 0.00 | 0.00 | 0.00 |
| Total | 99.72 | 100.00 | 100.23 |  | 100.59 | 100.36 | 100.15 |
| Mg# | 91.1 | 91.8 | 92.7 |  | 90.5 | 90.7 | 91.5 |

Mg# = 100 * Mg/ (Mg+Fe) (atomic numbers).

**Supplementary Information Table 3**

Mineral proportions and chemical compositions of the peridotite and carbonatitic xenoliths. Units are wt.% for major elements and ppm for trace elements.

|  | Peridotite xenolith (DLH-) | | |  | Carbonatite xenolith (DLH-) | | | | | | | | |
| --- | --- | --- | --- | --- | --- | --- | --- | --- | --- | --- | --- | --- | --- |
| Num. | 0606 | 0615 | 06119 |  | 0601 | 06102 | 0613 | 06108 | 06112 | 06113 | 06116 | 06117 | 06118 |
| **Carbonate and silicate mineral proportions (vol.%)** | | | | | | | | | | | | | |
| Carbonate | 5 | 0 | 0 |  | 98 | 90 | 50 | 80 | 97 | 98 | 70 | 50 | 98 |
| Silicate | 95 | 100 | 100 |  | 2 | 10 | 50 | 20 | 3 | 2 | 30 | 50 | 2 |
| **Chemical compositions** | | | | | | | | | | | | | |
| SiO_2_ | 46.9 | 44.0 | 43.9 |  | 0.94 | 6.01 | 21.69 | 4.05 | 5.62 | 9.24 | 13.5 | 12.14 | 3.98 |
| Al_2_O_3_ | 4.91 | 2.84 | 2.9 |  | 0.15 | 0.21 | 2.82 | 0.26 | 0.32 | 0.79 | 1.03 | 0.72 | 0.32 |
| TFeO | 7.02 | 8.17 | 7.94 |  | 0.16 | 1.80 | 5.29 | 1.17 | 1.31 | 2.17 | 3.74 | 3.69 | 1.12 |
| MgO | 32.9 | 41.0 | 41.8 |  | 0.45 | 2.42 | 10.84 | 1.51 | 3.33 | 4.52 | 6.93 | 7.56 | 2.08 |
| CaO | 5.69 | 2.13 | 1.56 |  | 54.9 | 49.3 | 33.2 | 51.4 | 48.8 | 45.4 | 41.2 | 41.6 | 50.9 |
| V | 161 | 62.4 | 64.1 |  | 2.79 | 12.3 | 66.9 | 5.17 | 13.9 | 17.6 | 48.2 | 52.6 | 14.3 |
| Cr | 3796 | 2632 | 5970 |  | 51.5 | 299 | 1159 | 257 | 352 | 483 | 1106 | 763 | 317 |
| Co | 149 | 158 | 161 |  | 3.29 | 28.8 | 43.7 | 15.2 | 16.0 | 27.9 | 30.2 | 50.2 | 23.2 |
| Ni | 1580 | 2033 | 2123 |  | 55.6 | 330 | 620 | 294 | 322 | 385 | 571 | 466 | 294 |
| Rb | 0.33 | 0.6 | 0.38 |  | 0.94 | 3.87 | 7.85 | 1.77 | 6.28 | 7.81 | 9.22 | 4.23 | 9.32 |
| Sr | 31.1 | 19.6 | 15.8 |  | 126 | 190 | 159 | 136 | 335 | 313 | 214 | 167 | 274 |
| Y | 5.69 | 2.24 | 1.38 |  | 6.17 | 0.19 | 6.48 | 0.22 | 0.36 | 1.72 | 2.75 | 0.56 | 1.15 |
| Zr | 11.8 | 6.05 | 6.77 |  | 8.98 | 1.14 | 15 | 1.84 | 1.36 | 2.12 | 3.17 | 4.98 | 1.77 |
| Nb | 0.61 | 1.31 | 0.69 |  | 0.25 | 0.27 | 0.97 | 0.37 | 0.76 | 0.69 | 0.51 | 1.92 | 0.42 |
| La | 1.57 | 0.78 | 0.46 |  | 3.85 | 0.15 | 1.36 | 0.19 | 0.2 | 0.94 | 1.57 | 0.40 | 0.36 |
| Ce | 3.09 | 1.68 | 1.23 |  | 0.32 | 0.31 | 3.75 | 0.21 | 0.27 | 0.97 | 1.86 | 0.42 | 0.50 |
| Pr | 0.50 | 0.21 | 0.16 |  | 0.56 | 0.04 | 0.57 | 0.038 | 0.042 | 0.15 | 0.20 | 0.054 | 0.067 |
| Nd | 2.57 | 0.99 | 0.84 |  | 2.45 | 0.19 | 2.94 | 0.16 | 0.18 | 0.68 | 0.74 | 0.23 | 0.34 |
| Sm | 0.81 | 0.26 | 0.24 |  | 0.49 | 0.043 | 0.86 | 0.043 | 0.06 | 0.16 | 0.15 | 0.06 | 0.096 |
| Eu | 0.27 | 0.097 | 0.08 |  | 0.15 | 0.016 | 0.29 | 0.013 | 0.017 | 0.048 | 0.051 | 0.022 | 0.034 |
| Gd | 0.94 | 0.29 | 0.25 |  | 0.61 | 0.037 | 0.93 | 0.041 | 0.053 | 0.19 | 0.18 | 0.058 | 0.10 |
| Tb | 0.16 | 0.055 | 0.043 |  | 0.097 | 0.007 | 0.17 | 0.007 | 0.008 | 0.034 | 0.033 | 0.011 | 0.017 |
| Dy | 0.97 | 0.37 | 0.26 |  | 0.63 | 0.037 | 1.07 | 0.04 | 0.046 | 0.23 | 0.24 | 0.069 | 0.12 |
| Ho | 0.20 | 0.078 | 0.05 |  | 0.14 | 0.008 | 0.23 | 0.006 | 0.008 | 0.049 | 0.06 | 0.018 | 0.027 |
| Er | 0.58 | 0.25 | 0.15 |  | 0.44 | 0.017 | 0.68 | 0.018 | 0.024 | 0.17 | 0.21 | 0.054 | 0.081 |
| Tm | 0.073 | 0.037 | 0.021 |  | 0.064 | 0.003 | 0.093 | 0.003 | 0.003 | 0.023 | 0.035 | 0.008 | 0.014 |
| Yb | 0.50 | 0.24 | 0.14 |  | 0.42 | 0.016 | 0.59 | 0.017 | 0.024 | 0.16 | 0.24 | 0.056 | 0.084 |
| Lu | 0.073 | 0.039 | 0.023 |  | 0.074 | 0.003 | 0.094 | 0.004 | 0.005 | 0.025 | 0.044 | 0.012 | 0.016 |
| Hf | 0.41 | 0.16 | 0.18 |  | 0.034 | 0.025 | 0.36 | 0.027 | 0.034 | 0.051 | 0.042 | 0.048 | 0.039 |
| Ta | 0.17 | 0.19 | 0.15 |  | 0.038 | 0.039 | 0.11 | 0.028 | 0.029 | 0.033 | 0.041 | 0.038 | 0.03 |
| Pb | 0.42 | 0.17 | 0.20 |  | 0.12 | 0.14 | 0.52 | 0.079 | 0.16 | 0.22 | 0.17 | 0.085 | 0.11 |
| Th | 0.055 | 0.11 | 0.048 |  | 0.024 | 0.018 | 0.14 | 0.009 | 0.012 | 0.022 | 0.14 | 0.03 | 0.021 |
| U | 0.051 | 0.028 | 0.013 |  | 0.43 | 0.25 | 0.30 | 0.33 | 0.58 | 0.59 | 0.39 | 0.35 | 0.60 |

**Supplementary Information Table 4**

Blank, detection limits and international rock standards analyzed by ICP-MS

|  | Blank | DL |  | AGV-2 | |  | BHVO-2 | |  | BCR-2 | |  | RGM-2 | |
| --- | --- | --- | --- | --- | --- | --- | --- | --- | --- | --- | --- | --- | --- | --- |
|  |  |  |  | Ref | Mea |  | Ref | Mea |  | Ref | Mea |  | Ref | Mea |
| V | 0.34 | 0.134 |  | 120 | 112 |  | 317 | 319 |  | 416 | 423 |  | 13.0 | 11.9 |
| Cr | 0.049 | 0.106 |  | 16.0 | 15.4 |  | 280 | 288 |  | 16.5 | 16.0 |  | 5.90 | 2.86 |
| Co | 0.045 | 0.040 |  | 16.0 | 15.6 |  | 45.0 | 44.6 |  | 37.0 | 37.8 |  | 2.00 | 2.04 |
| Ni | 0.14 | 0.038 |  | 20.0 | 19.3 |  | 119 | 126 |  | 13.0 | 13.3 |  | 5.20 | 2.32 |
| Rb | 0.023 | 0.011 |  | 66.3 | 67.1 |  | 9.11 | 9.18 |  | 46.9 | 47.5 |  | 150 | 149 |
| Sr | 0.032 | 0.174 |  | 661 | 655 |  | 396 | 398 |  | 340 | 347 |  | 108 | 105 |
| Y | 0.011 | 0.007 |  | 20.0 | 19.6 |  | 26.0 | 26.1 |  | 37.0 | 36.8 |  | 23.2 | 23.6 |
| Zr | 0.034 | 0.072 |  | 230 | 230 |  | 172 | 164 |  | 184 | 183 |  | 220 | 230 |
| Nb | 0.011 | 0.004 |  | 14.5 | 14.0 |  | 18.1 | 18.7 |  | 12.6 | 12.7 |  | 9.30 | 9.19 |
| La | 0.009 | 0.005 |  | 37.9 | 37.8 |  | 15.2 | 15.4 |  | 24.9 | 25.6 |  | 24.0 | 23.3 |
| Ce | 0.012 | 0.013 |  | 68.6 | 69.0 |  | 37.5 | 37.6 |  | 52.9 | 53.6 |  | 47.0 | 45.9 |
| Pr | 0.007 | 0.002 |  | 7.84 | 8.00 |  | 5.35 | 5.26 |  | 6.70 | 6.80 |  | 5.36 | 5.20 |
| Nd | 0.018 | 0.012 |  | 30.5 | 30.0 |  | 24.5 | 24.4 |  | 28.7 | 29.1 |  | 19.0 | 19.2 |
| Sm | 0.013 | 0.021 |  | 5.49 | 5.60 |  | 6.07 | 6.12 |  | 6.58 | 6.82 |  | 4.30 | 3.94 |
| Eu | 0.007 | 0.006 |  | 1.54 | 1.49 |  | 2.07 | 2.14 |  | 1.96 | 1.99 |  | 0.66 | 0.62 |
| Gd | 0.013 | 0.017 |  | 4.52 | 4.62 |  | 6.24 | 6.21 |  | 6.75 | 6.76 |  | 3.70 | 3.63 |
| Tb | 0.007 | 0.001 |  | 0.64 | 0.64 |  | 0.92 | 0.95 |  | 1.07 | 1.10 |  | 0.66 | 0.61 |
| Dy | 0.012 | 0.010 |  | 3.47 | 3.56 |  | 5.31 | 5.38 |  | 6.41 | 6.62 |  | 4.10 | 3.76 |
| Ho | 0.008 | 0.002 |  | 0.65 | 0.66 |  | 0.98 | 0.98 |  | 1.28 | 1.34 |  | 0.82 | 0.76 |
| Er | 0.009 | 0.006 |  | 1.81 | 1.76 |  | 2.54 | 2.53 |  | 3.66 | 3.74 |  | 2.35 | 2.33 |
| Tm | 0.007 | 0.002 |  | 0.26 | 0.26 |  | 0.33 | 0.33 |  | 0.54 | 0.55 |  | 0.37 | 0.36 |
| Yb | 0.011 | 0.010 |  | 1.62 | 1.62 |  | 2.00 | 2.01 |  | 3.38 | 3.44 |  | 2.60 | 2.53 |
| Lu | 0.008 | 0.003 |  | 0.25 | 0.25 |  | 0.27 | 0.27 |  | 0.50 | 0.52 |  | 0.40 | 0.39 |
| Hf | 0.011 | 0.014 |  | 5.00 | 5.13 |  | 4.36 | 4.41 |  | 4.90 | 5.02 |  | 6.20 | 5.91 |
| Ta | 0.009 | 0.006 |  | 0.87 | 0.87 |  | 1.14 | 1.17 |  | 0.78 | 0.80 |  | 0.95 | 0.91 |
| Pb | 0.016 | 0.027 |  | 13.2 | 13.3 |  | 1.60 | 1.64 |  | 11.0 | 10.5 |  | 19.3 | 19.7 |
| Th | 0.007 | 0.005 |  | 6.10 | 6.15 |  | 1.22 | 1.24 |  | 5.70 | 6.09 |  | 15.1 | 14.7 |
| U | 0.006 | 0.004 |  | 1.86 | 1.90 |  | 0.40 | 0.42 |  | 1.69 | 1.75 |  | 5.80 | 5.68 |
| * Concentrations are reported in ppm for the rock standards, and ppb for Blank and detection limit (DL). Ref = reference value; Mea = measured value. | | | | | | | | | | | | | | |

**Figures**

**Supplementary Information Fig 1**

Tectonic sketch map of the Inner Mongolia–Daxinganling Orogenic Belt modified from Xiao and Windley (2003).


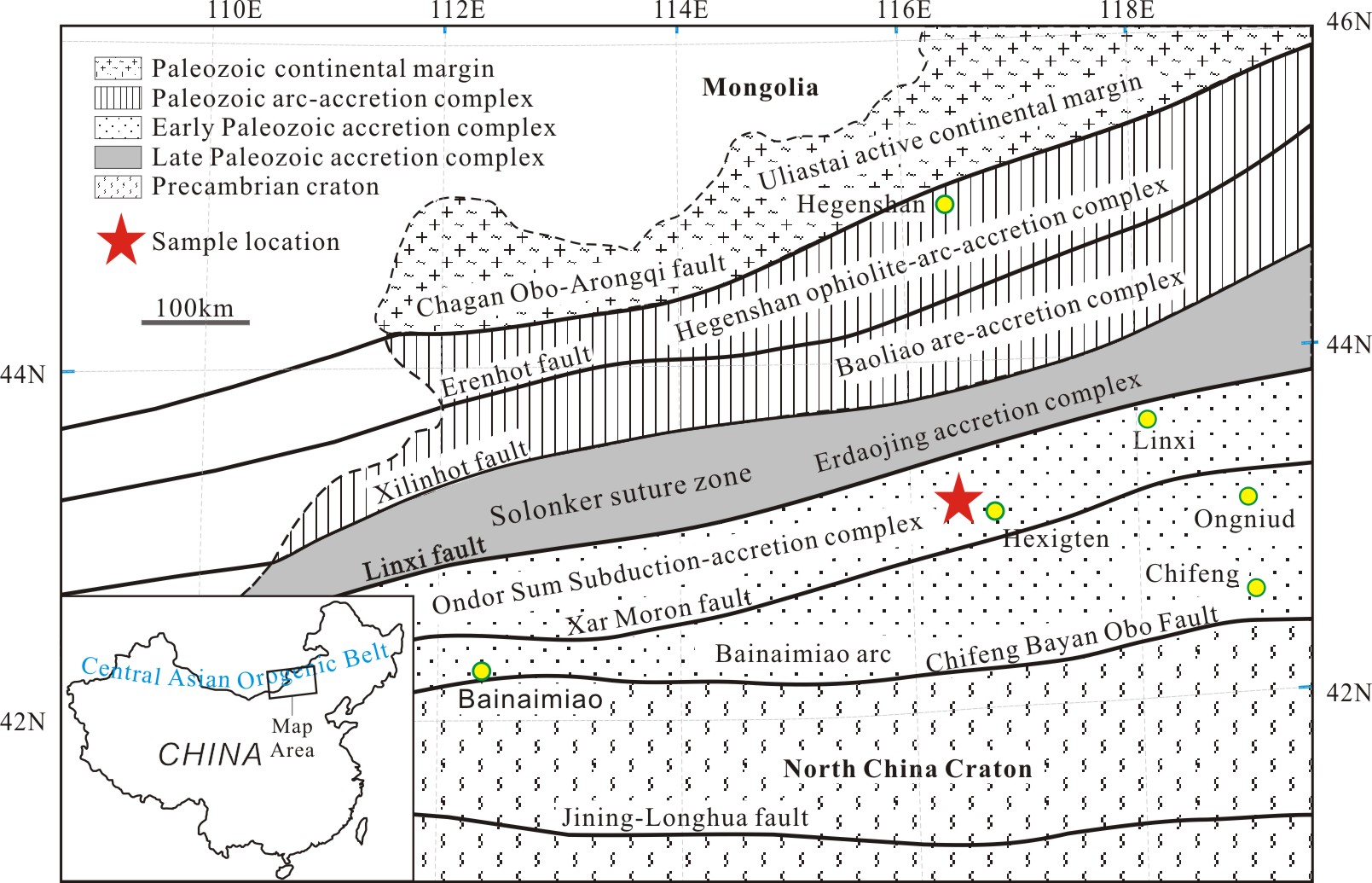


**Supplementary Information Fig 2**

(a) Raman shift of diamond from the carbonatitic xenolith; (b) Raman shift of a zircon inclusion found in one moissanite grain, (c) Raman shift of ordered graphite from the carbonatitic xenolith; (d) Raman shift of disordered graphite from the carbonatitic xenolith.


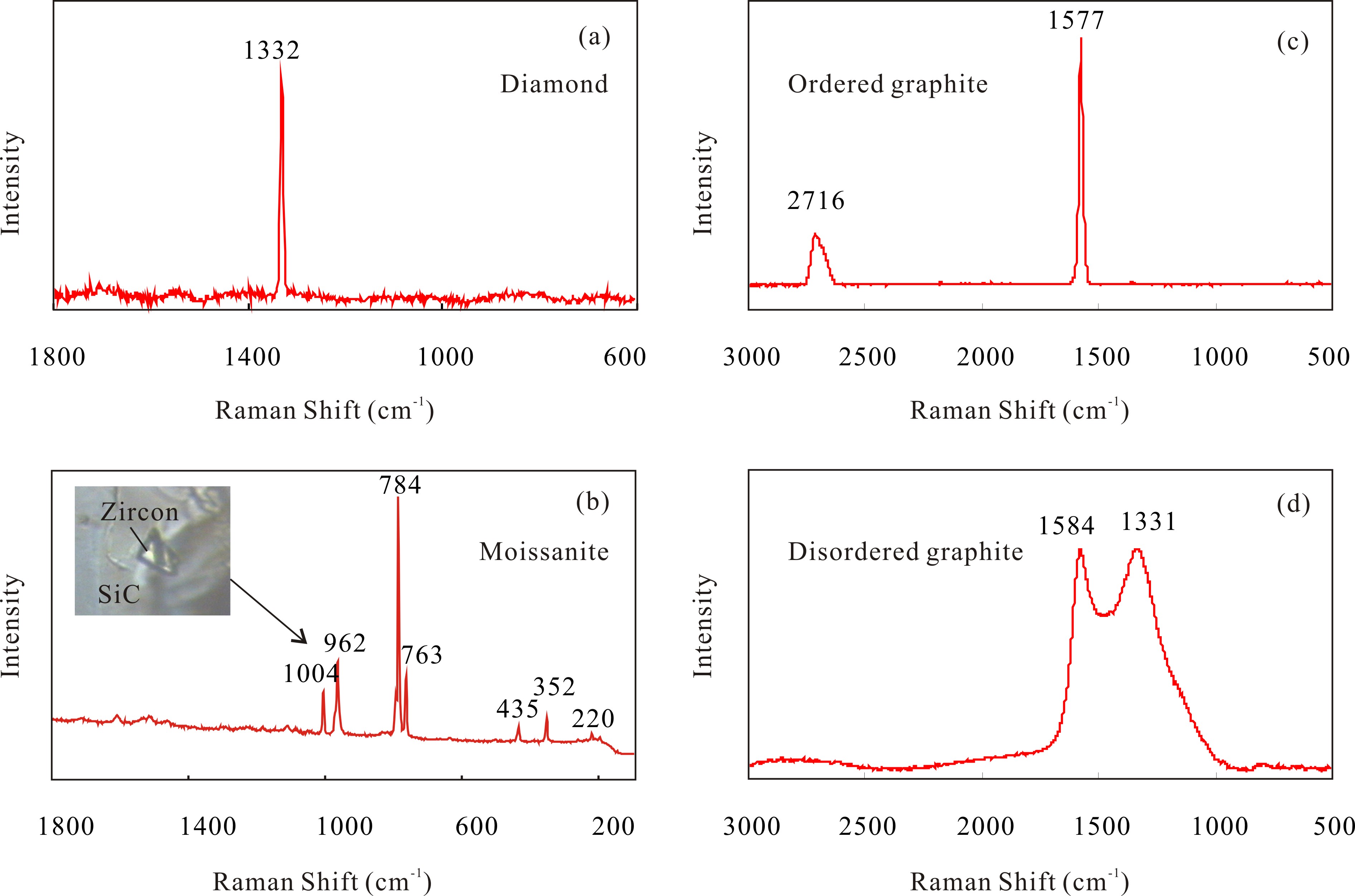

Supplement: Supplementary Information [file srep11547-s1.doc]
